# Analytical Studies on the Compressive Properties of Mortise–Tenon Interlocking Grouted Masonry

**DOI:** 10.3390/ma19030522

**Published:** 2026-01-28

**Authors:** Shugang Yu, Zhongmin Han, Kaiwei Liu, Kai Zhang, Yichen Yang, Juntao Zhu

**Affiliations:** 1 School of Civil Engineering, Zhengzhou University, Zhengzhou 450001, China; 2 Henan Dajian Architectural Design Co., Ltd., Zhengzhou 450001, China; 13939062893@163.com

**Keywords:** masonry, mortise–tenon interlocking design, numerical simulation, analytical studies

## Abstract

This paper proposes a novel mortise-and-tenon grouted masonry (MTGM) structure to enhance the mechanical performance and engineering applicability of masonry. The axial and eccentric compressive behavior of the system was systematically investigated through experimental testing and numerical simulation. A refined three-dimensional finite element model, developed in DIANA, effectively accounted for material nonlinearity and interfacial contact, with its high accuracy confirmed by experimental results. The parametric analysis of 52 numerical models elucidated the influence of block strength, core material type, wall thickness, steel fiber content, and geometric ratios on the compressive strength, deformation capacity, and failure modes. The results demonstrate that using steel fiber-reinforced concrete (SFRC) as the core filling material significantly enhances ductility and toughness; an SFRC content of 1.6% increased the ultimate strain by approximately 37%. Furthermore, increasing the eccentricity from 0.1 to 0.3 led to an average 40% reduction in load-bearing capacity. Theoretical analysis led to the derivation of calculation formulae relating to key axial compression parameters. Furthermore, a stress–strain constitutive relationship suitable for MTGM was established, featuring a parabolic ascending branch and a linear descending branch (R^2^ = 0.992). For eccentric compression, a practical design method was developed based on the plane section assumption, which demonstrated superior predictive accuracy compared to existing code provisions. This study provides a reliable theoretical foundation and practical computational tools for the structural design and application of MTGM.

## 1. Introduction

Masonry structures have long held a significant position in the construction industry due to their outstanding cost-effectiveness and superior sound insulation and fire-resistant properties [1,2,3,4]. Compared to other modern materials, they present a lower technical barrier while providing effective thermal and acoustic insulation, making them particularly suitable for low-to-mid-rise buildings and an indispensable structural type in engineering practice [5]. However, traditional masonry relies on mortar bonding, which suffers from low construction efficiency, significant on-site material waste, and high demands in terms of skilled labor [6,7], limiting the further development of masonry’s structural performance.

Core grouting technology, as an effective approach to enhance masonry integrity and bonding performance, has become a current research focus. Through grouting operations, the grout tightly bonds blocks together, significantly improving walls’ load-bearing capacities [8]. Currently, many countries widely adopt internal grouting techniques in traditional concrete block masonry to enhance the overall load-bearing capacity of walls against axial and lateral forces [9]. Regarding the performance regulation of grouted masonry, the individual strength of concrete blocks and the inherent strength of the grout serve as foundational conditions. Compressive strength does not depend on a single material but rather on the rational matching of both components; stronger materials can directly elevate the overall structural strength [10,11]. Furthermore, parameters such as the grout filling rate of blocks, the height-to-thickness ratio and length-to-thickness ratio of specimens, and mortar thickness critically influence the mechanical properties of grouted masonry [12]. Researchers have conducted in-depth investigations into grouted masonry through experiments and numerical simulations, yielding significant findings. The stress-transferring capacity of mortar should not be equated with its strength. To systematically analyze the compressive behavior of different masonry types, Ouyang et al. [13] proposed a unified theory based on the internal structure of I-beams. By simulating geometric behavior through equivalent frames and material properties via constitutive relationships, they successfully predicted the compressive response of various masonry types, providing a framework for masonry mechanical analysis. Sathurshan et al. [14] experimentally evaluated the compressive strength, failure mechanisms, and the stress–strain behavior of five masonry units under grouted and ungrouted conditions. The results indicated that grouting significantly enhances masonry compressive strength and stiffness, with grouted specimens achieving 57–64% of block strength. The study also validated the applicability of existing analytical models for predicting such structures. Mohammad Bolhassani et al. [15] further developed a micro-modeling approach based on concrete damage–plasticity models and cohesive interface behavior, effectively simulating failure mechanisms in hollow, partially grouted, and fully grouted masonry. Calderón et al. [16] utilized the nonlinear finite element software DIANA to simulate the mechanical response of unreinforced grouted masonry under axial compression. The results demonstrated high agreement between simulations and experimental data, confirming the software’s applicability in such analyses.

Meanwhile, innovation in connection methods represents another critical pathway for enhancing structural performance. Among these, mortarless interlocking blocks optimize interfacial force transfer mechanisms through physical interlocking, offering both construction convenience and cost-effectiveness. They have been widely adopted in structures such as residential buildings, dams, and piers [17,18]. The mortise-and-tenon joint in traditional Chinese timber structures, as a classic interlocking form, offers new insights for modern prefabricated masonry development due to its excellent seismic performance. As semi-rigid connections, mortise-and-tenon joints possess an inherent gap between the tenon and mortise. Internal forces are transmitted through normal compression and tangential friction, while energy dissipation occurs via bending deformation and frictional sliding [19]. The connection form and geometric parameters of the mortise-and-tenon joints have a decisive influence on their mechanical behavior. According to their structure, it can be classified into several types, including a straight mortise–tenon frame, dovetail mortise–tenon frames, penetrated mortise–tenon frames, and half mortise–tenon frames [20]. Chen et al. [21] investigated the seismic performance of spatial and planar hoop–head mortise–tenon joints through low-cycle reversed loading tests, revealing that all failures occurred within the joint area and that the hysteresis curves exhibited a distinct pinched, reverse Z-shape. The study demonstrated that increasing the tenon width moderately improved positive loading stiffness and peak moment. Zhang et al. [22] examined the effect of looseness-induced inclination on the seismic behavior of mortise–tenon joints through cyclic tests and multi-scale finite element modeling. The results indicated that increased inclination rotation significantly reduced the hysteretic loop area, ultimate moment-resisting capacity, initial elastic stiffness, and ductility, while also making negative initial slippage and pinching effects more pronounced. Ren et al. [23] experimentally and theoretically analyzed the rotational performance of cross-shaped joints with dowels, demonstrating that the addition of a dowel effectively enhanced the joint’s bearing capacity, rotational ductility, and resistance to tenon extraction. The hysteresis curves displayed a Z-shape with pinching and sliding effects, and the proposed theoretical model accurately predicted the moment–rotation relationship. It was also found that a dowel size between 10 and 30 mm optimized the joint’s rotation capacity. Wu et al. [24] experimentally studied the lateral performance of half mortise–tenon frames, considering various support conditions and analyzing the effects of column height and diameter. Xie et al. [25] investigated the lateral behavior of a straight mortise–tenon frame via low-cycle reversed loading tests, highlighting the significant influence of both vertical loads and column height on the performance of traditional Chinese timber frames.

Parallel to dry-stack interlocking, the integration of grouting technology with novel block systems presents another promising direction. Ma et al. [26] experimentally demonstrated that grouting into holes perpendicular to interlocking blocks creates an effective locking mechanism. This mechanism not only eliminates initial gaps between blocks but also significantly enhances the overall stability of the wall. Building upon this general concept, the mortise–tenon grouting masonry (MTGM) system presented in this study was developed. MTGM integrates a physical mortise-and-tenon interlocking mechanism with core grouting technology, thereby combining the convenience of dry assembly with the integral reinforcement provided by grout. While Ma et al.’s work validates the grouting principle for earth-based blocks, their connection is grout dependent, whereas MTGM employs a synergistic hybrid system where mechanical interlocking and grouting complement each other.

However, for such composite systems, a systematic understanding of how macroscopic mechanical properties are influenced by the parameters of the mortise-and-tenon structure and the grouting technique is lacking. To address this gap, this research is conducted through three integrated stages. (1) Modeling and Validation: Developing a high-fidelity 3D nonlinear finite element model in DIANA that accurately captures the complex material and interfacial behavior of MTGM, followed by rigorous experimental validation. (2) Systematic Parametric Analysis: Executing an extensive numerical study comprising 19 axial and 33 eccentric compression models to quantify the influence of the aforementioned parameters on strength, deformation, ductility, and failure modes. (3) Theoretical Modeling and Design Method Development: Deriving, based on the numerical results and mechanical principles, new analytical tools, including calculation formulas for axial compression parameters, a complete stress–strain constitutive model, and a practical design method for eccentric load-bearing capacity. This research aims to develop predictive calculation tools and a design-oriented constitutive relationship for the MTGM system, thereby refining its theoretical framework for engineering application.

## 2. FEM Frameworks

### 2.1. FEM Model

**Figure 1:**
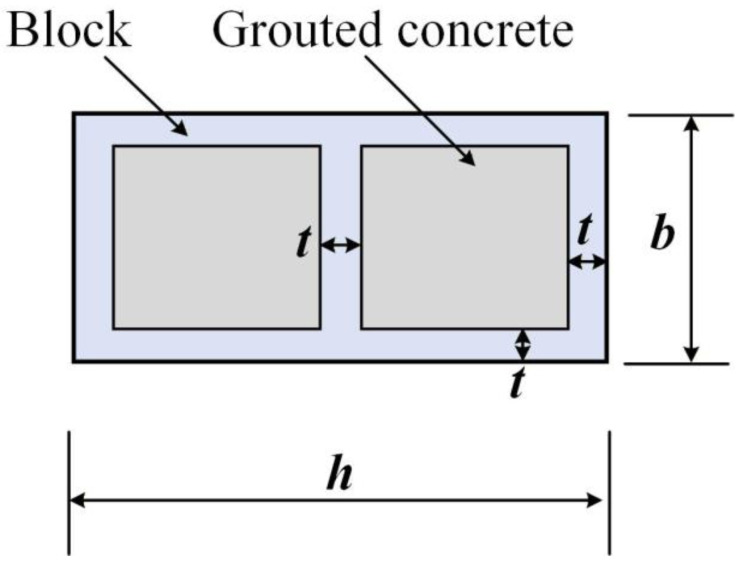
Schematic diagram of masonry cross-sectional structure.

Based on the nonlinear finite element software DIANA FEA 10.6 [27], a three-dimensional solid modeling method was adopted to establish the model of the MTGM. The structure and dimensions of all simulation numerical models were kept consistent, with their height × thickness × width (*h* × *t* × *b*) being 600 mm × 400 mm × 200 mm. The basic structure of this mortise-and-tenon grouted masonry (MTGM) is as follows: the unit block is composed of two C-shaped blocks and one I-shaped block, where the vertical edges of the I-shaped block are clamped to the inner side of the vertical edges of the C-shaped blocks; the blocks in each layer are assembled layer by layer using the same connection process to form a box-shaped hollow block structure; and after pouring grouted concrete into the block holes, a complete MTGM is thus formed. The cross-sectional diagram of masonry is shown in Figure 1.

#### 2.1.1. Material Constitutive Model

The total strain-based cracking model was adopted to simulate the blocks and grouted concrete. The compressive and tensile stress–strain relationships of the blocks and the ordinary grouted concrete were all characterized by a fracture energy-based parabolic model and an exponential model, respectively [27]. The tensile fracture energy (*G_f_*) and compressive fracture energy (*G_c_*) of the concrete are expressed as follows [28]:

(1) $$G_{f}=0.073f_{c}^{0.18}$$

(2) $$G_{c}=250G_{f}$$

where *f*_c_ is the axial compressive strength of concrete.

For concrete under compression, the elastic strain *ε*_0_, peak strain *ε*_c_, and ultimate strain *ε*_u_ are, respectively, given as Equations (3)–(5):

(3) $$\varepsilon_{0}=\frac{f_{c}}{3E}$$

(4) $$\varepsilon_{c}=\frac{5f_{c}}{3E}$$

(5) $$\varepsilon_{u}=\frac{5f_{c}}{3E}+\frac{3G_{c}}{2hf_{c}}$$

where *E* is the elastic modulus of concrete.

For the grouting of steel fiber-reinforced concrete (SFRC), its stress–strain relationship under compression still adopts the fracture energy-based parabolic model, with the enhancement effect of steel fibers on compressive strength taken into account, while its stress–strain relationship under tension adopts the RILEM model [29]. Among these, the compressive fracture energy of SFRC (*G*_sc_) is given as follows:

(6) $$G_{sc}=18.25f_{sc}^{0.18}$$

where *f*_sc_ is the compressive strength of SFRC, which can be calculated by Equation (7):

(7) $$f_{\mathrm{sc}}=\left(1+\alpha_{c}n_{f}\frac{l_{f}}{d_{f}}\right)f_{c}$$

where *n_f_*, *l_f_*, and *d_f_* are the volume content of steel fibers, the length of steel fibers, and the diameter of steel fibers, respectively. The coefficient *α_c_* is determined through experiments, and its values are presented in Table 1 [30].

The compressive peak strain of SFRC can be expressed as Equation (8):

(8) $$\varepsilon_{\mathrm{sc}}=\left(1+\beta n_{f}\frac{l_{f}}{d_{f}}\right)\varepsilon_{c}$$

where the coefficient *β* is determined through experiments, and its values are presented in Table 1 [30].

In addition, the compressive ultimate strain of SFRC can be calculated by substituting Equations (7) and (8) into Equation (5). Based on the Standard for Design of Steel Fiber-Reinforced Concrete Structures [31], the uniaxial tensile strength of SFRC (*f*_st_) is given as Equation (9):

(9) $$f_{st}=\left(1+\alpha_{t}n_{f}\frac{l_{f}}{d_{f}}\right)f_{t}$$

where *f*_t_ is the tensile strength of concrete, the coefficient *α_t_* is determined through experiments, and its values are presented in Table 1.

The uniaxial residual tensile strength of SFRC (*f_sf_*) can be expressed as Equation (10):

(10) $$f_{sf}=0.43f_{t}n_{f}\frac{l_{f}}{d_{f}}F_{be}$$

where *F_be_* represents the shape factor of steel fibers, and a value of 1.0 is adopted for this study [32].

The uniaxial ultimate tensile strength of SFRC (*f*_stu_) can be expressed as Equation (11):

(11) $$f_{\mathrm{stu}}=0.148f_{R,4}$$

where *f_R_*_,4_ is the ultimate flexural tensile strength corresponding to the CMOD curve.

#### 2.1.2. Finite Element Parameter Settings

Hexahedral solid elements (HX24L) with an element size of 20 mm were used for the blocks, grouted concrete, and steel bearing plates, where a 20 mm thick plate was arranged on both the top and bottom surfaces of the masonry. Surface-to-surface interface elements (Q24IF) were employed for the contact interfaces between blocks and grouted concrete, as well as between the steel bearing plates and masonry. The bottom surface of the support steel plate was constrained in three translational degrees of freedom (*u_x_*, *u_y_*, and *u_z_*), while a displacement load was applied to the top surface of the loading plate with a load step of 0.04 mm per step.

For the interface between the steel plate and concrete, linear elastic contact was adopted. The normal contact stiffness was set to *E_p-c_*/*l_e_*, where *E_p-c_* denotes the average elastic modulus of the steel plate and concrete, and *l_e_* represents the average size of elements near the interface, while the tangential contact stiffness was 1/10 of the normal stiffness. No bond slip was considered between the blocks and grouted concrete, and a tied constraint was applied instead. For the normal direction between blocks, linear elastic contact, whose stiffness was set to *E_c_*/*l_e_*, was used, while a Coulomb friction contact, a dry connection, was adopted for the tangential direction. The cohesion, friction angle, and dilation angles were 0.01 MPa, 0.785 rad, and 0.1 rad, respectively. The contact settings for blocks in different layers were consistent with the above specifications.

In total, 19 groups of models were prepared to investigate the axial compression behavior of the MTGM. The parameters considered included block concrete strength (C30, C40, and C50), block wall thickness (10, 20, 30, and 40 mm), the type and strength of grouted concrete (ordinary concrete: C30, C40, C50; SFRC: CF30, CF40, and CF50), and the steel fiber volume fraction (1%, 1.3%, 1.6%). In addition, 33 sets of models were designed to simulate eccentric compression. Among them, the concrete strength grade of the blocks was C30, the wall thickness was 20 mm, and the masonry thickness was 400 mm. The variable parameters included eccentricity (*e*/*h* = 0.1, 0.2, and 0.3), the type and strength of core-filled concrete (normal concrete: C30; SFRC: CF30, CF40, and CF50), the steel fiber content (1%, 1.3%, and 1.6%), the width-to-thickness ratio (*b*/*h* = 0.4, 0.5, 0.63, and 0.75, corresponding to widths of 160 mm, 200 mm, 250 mm, and 300 mm), and the height-to-thickness ratio (*H*/*h* = 1.5, 2.0, and 3.0, corresponding to heights of 600 mm, 800 mm, and 1200 mm).

### 2.2. Model Validation

#### 2.2.1. Failure Patterns

**Figure 2:**
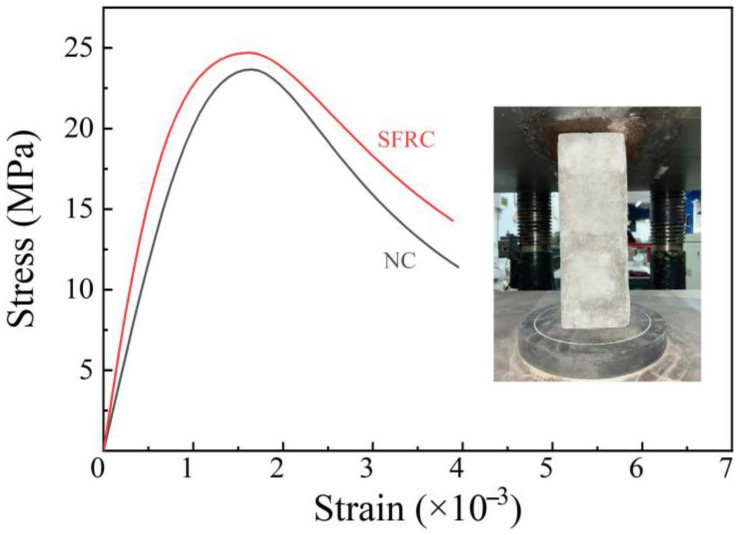
Stress–strain curves for normal concrete and steel fiber-reinforced concrete.

The materials used to fabricate the validation specimens are specified as follows. The concrete for blocks and grout was cast on-site with a mix proportion (by weight) of cement:sand:aggregate:water = 1:2.03:3.76:0.6. P.O 42.5, natural river sand (2–3 mm), coarse gravel (8–12 mm), and tap water were used. For the steel fiber-reinforced concrete (SFRC) grout, hooked-end steel fibers with a length of 30 mm and a diameter of 0.6 mm were incorporated at specified volume fractions. To facilitate understanding of the material’s properties, Figure 2 provides a stress–strain diagram.

**Figure 3:**
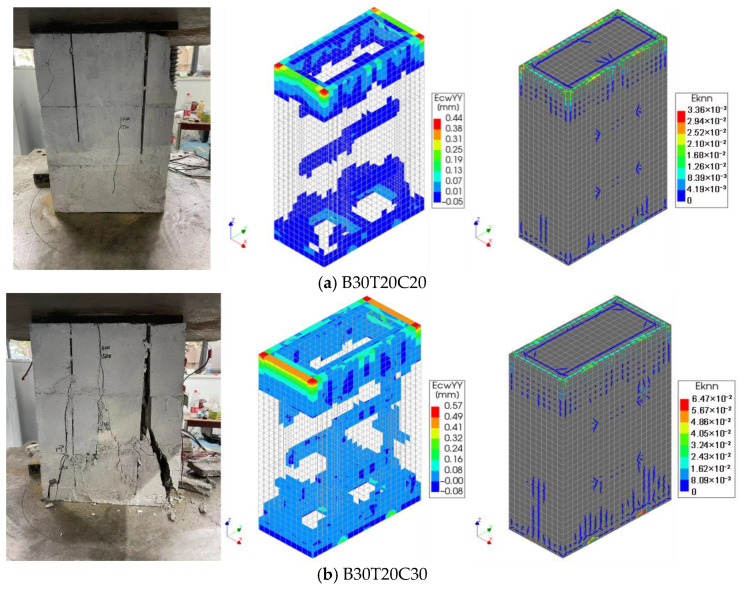

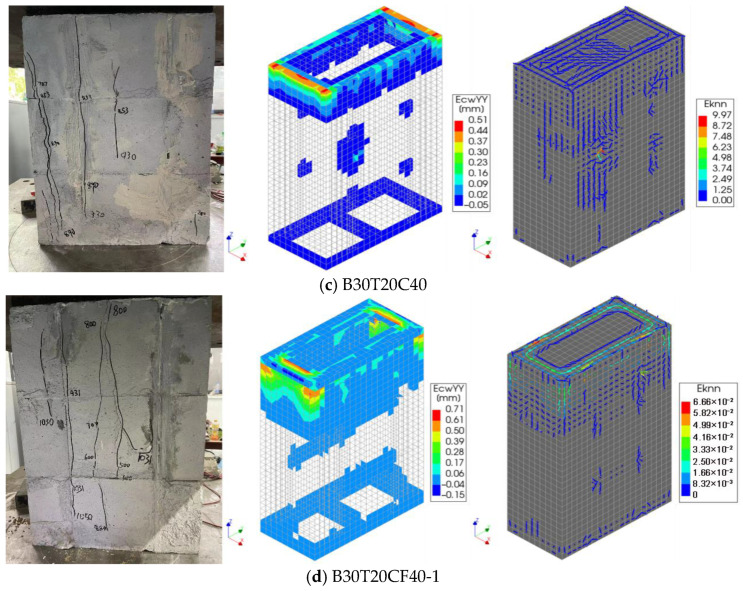
Comparison between experimental and simulated results of axial compression failure modes of masonry.

To validate the finite element model, four different MTGM specimens were designed, and three specimens for each design were tested. The results from each set of three specimens were averaged to minimize random errors. The specimen dimensions (600 mm × 400 mm × 200 mm) were chosen to represent a typical masonry prism in structural testing, balancing the need to reflect full-scale behavior with laboratory constraints. This scale is considered representative for assessing the global compressive behavior of masonry systems, while remaining manageable in terms of fabrication, handling, and testing under laboratory conditions. Figure 3 compares the experimental and simulated failure modes of masonry under axial compression. In “BxTyCz”, x represents the block strength grade, y represents the wall thickness, and z represents the strength of the grouting concrete. In “CFm-n”, m represents the strength of the grouting steel fiber-reinforced concrete and n represents the content of steel fibers. The experimental and simulated crack patterns show good overall agreement. The main damage is concentrated in the top plate and adjacent regions, where direct contact with the steel bearing plates leads to stress concentration. In addition, the simulated results clearly show vertical interface cracks at the mortise-and-tenon joints, which correspond closely to the experimental observations. Vertical micro-cracks were observed along the interface, particularly near mortise–tenon regions, indicating bond degradation under load. Stress transfer initially relied on friction, transitioning to aggregate interlock as the cracking progressed—a behavior enhanced by steel fiber bridging in SFRC-filled specimens. These patterns align with the simulated friction-based contact model, supporting the use of Coulomb friction parameters in the analysis. Therefore, the nonlinear finite element model developed in this study accurately reproduces the axial compression failure behavior of the MTGM.

#### 2.2.2. Load–Displacement Curve Verification

**Figure 4:**
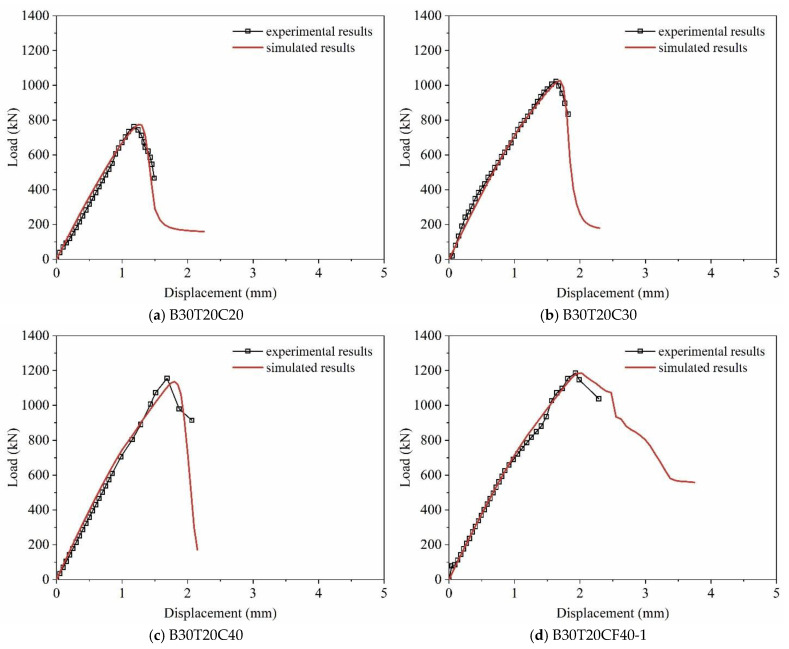
Comparison between experimental and simulated results of load–displacement curves of masonry under axial compression.

Figure 4 compares the experimental and simulated load–displacement curves of the masonry under axial compression. The experimental value is the average of three parallel specimens. The experimental and simulated load–displacement curves of the masonry under axial compression show good agreement. For the curve peak points, the mean value and coefficient of variation in the simulated-to-experimental peak load ratio are 0.96 and 0.07, respectively, while those of the peak displacement ratio are 1.01 and 0.10, respectively. The high consistency in both the linear elastic stiffness, the peak state (load and displacement), and the post-peak softening trend across all four specimen types confirms that the developed model not only captures the ultimate capacity but also reliably replicates the complete load–deformation response and material degradation process of the MTGM system. This comprehensive validation provides a solid foundation for the subsequent parametric numerical analysis. Minor discrepancies are due to inherent material variability, idealized interfacial modeling, and experimental imperfections.

## 3. FEM Simulation Results and Discussion on Axial Compression

### 3.1. Stress–Strain Curve

**Figure 5:**
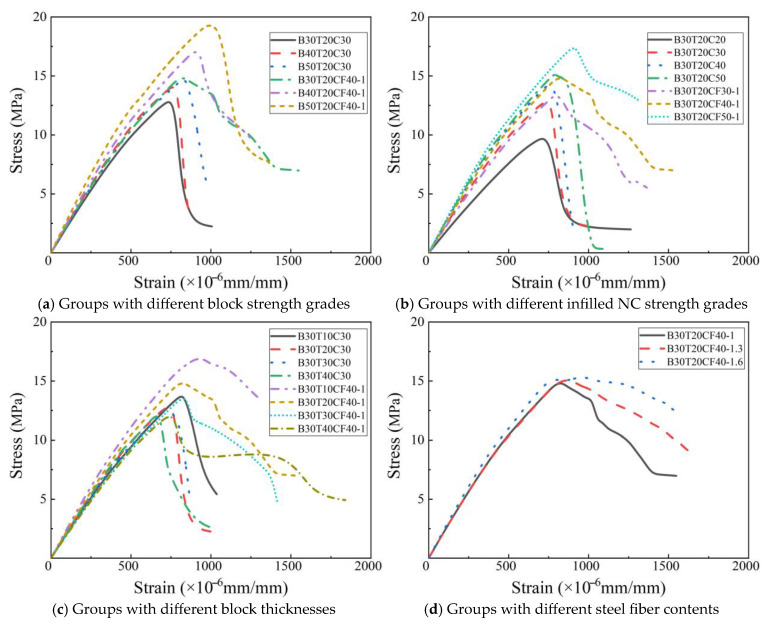
The stress–strain curves of models in different parameter groups.

The compressive stress–strain curves of the different masonry are shown in Figure 5. The compressive stress–strain curve of masonry can be divided into two stages. In the ascending stage, the curve is linear; with the continuous application of load, the growth rate of strain accelerates gradually. In the descending stage, the masonry fails after reaching the compressive strength, the stress decreases rapidly with the increase in strain, and the masonry thus loses its bearing capacity. It is worth noting that when core-filled steel fiber-reinforced concrete (SFRC) is adopted, the slope of the descending stage of the masonry becomes gentler, which indicates that core-filled SFRC is beneficial to delaying the brittle failure process of the masonry. From Figure 5a, it can be observed that with the increase in block strength, the slope of the masonry stress–strain curve increases gradually, and its peak strain and peak stress also increase accordingly. Figure 5b illustrates that with the increase in the strength of core-filled concrete, the slope of the masonry stress–strain curve, peak strain, and compressive strength all increase gradually. As depicted in Figure 5c, with the increase in the block wall thickness, both the peak strain and compressive strength of the masonry decrease. It is notable that the slope of the stress–strain curve of masonry filled with normal concrete (NC) shows almost no change, while the slope of the stress–strain curve of core-filled SFRC masonry decreases with the increase in block wall thickness. This phenomenon is mainly due to the fact that the strength of infilled NC is C30, which is consistent with the block strength, while the strength of infilled SFRC is C40. When the wall thickness increases, the proportion of higher-strength infilled SFRC decreases, which in turn leads to a decrease in stiffness. As shown in Figure 5d, increasing the steel fiber content in core-filled SFRC has a limited improvement effect on the slope of the compressive stress–strain curve, compressive strength, and peak strain of the masonry, but it has a significant effect with regard to reducing the slope of the descending stage of the curve and increasing the ultimate strain. This indicates that increasing the steel fiber content of core-filled SFRC within a certain range is beneficial to improving the toughness and ductility of mortise–tenon core-filled masonry.

### 3.2. Peak Strain

From Figure 5a, it can be seen that the peak strain of masonry increases approximately linearly with the increase in block strength grade. In addition, the peak strain of core-filled SFRC masonry is generally higher than that of core-filled NC masonry, but the difference between the two does not change significantly with the increase in block strength. As shown in Figure 5b, the peak strain of masonry increases with the improvement of core-filled concrete strength grade, among which the peak strain of core-filled SFRC masonry increases more significantly. This is because steel fibers can further unleash the deformation potential of high-strength concrete through crack resistance, toughening, and improving interface performance [33]. It is observed from Figure 5c that the peak strain of masonry decreases gradually with the increase in block wall thickness, and the decreasing laws of peak strain for core-filled NC and core-filled SFRC masonry are basically consistent. The larger the block wall thickness, the more significant the improvement of its cross-sectional compressive stiffness, which causes the block to bear a larger proportion of the load under compression, while the load contribution ratio of core-filled concrete (NC or SFRC) is relatively reduced. As indicated in Figure 5d, the peak strain of masonry increases slightly with the increase in steel fiber content in core-filled SFRC. This is mainly because steel fibers can effectively span microcracks and prevent their propagation, forcing cracks to either bypass the fibers or pull them out to propagate further [34]. This process consumes more energy, thus manifesting as higher peak strain.

In summary, the compressive peak strain of masonry is positively correlated with block strength, core-filled concrete strength, and filling ratio. Based on the experimental data and numerical fitting, the peak strain of the core-filled masonry can be expressed as a function of the peak strain of the masonry blocks and the peak strain of the core-filled concrete:

(12) $$\varepsilon_{m}=C\varepsilon_{b}+D\gamma \sqrt{\varepsilon_{b}\varepsilon_{sc}}$$

where *ε_m_* is the peak strain of MTGM, *ε_b_* is the peak strain of block concrete, *ε*_sc_ is the peak strain of SFRC, *γ* is the filling ratio of core-filled concrete, *γ* = *A*_0_/*A*, *A* is the cross-sectional area, and *A*_0_ is the grouting area.

Based on the test data and numerical fitting, the coefficients *C* and *D* in Equation (12) are 0.291 and 0.254, respectively, with the coefficient of determination R^2^ = 0.83. Therefore, Equation (12) can be further expressed as follows:

(13) $$\varepsilon_{m}=0.291\varepsilon_{b}+0.254\gamma \sqrt{\varepsilon_{b}\varepsilon_{sc}}$$

### 3.3. Compressive Strength

As shown in Figure 5a, the compressive strength of masonry increases with the improvement of block strength grade. Notably, compared with core-filled NC masonry, the compressive strength of core-filled SFRC masonry exhibits a more significant growth rate. This may be attributed to the stronger confining effect of the block on the SFRC in the core area [35], thereby enabling the masonry to achieve a more obvious improvement in compressive strength. As indicated in Figure 5b, the compressive strength of masonry increases with the increase in core-filled concrete strength. Similarly, the improvement rate of the compressive strength of the core-filled SFRC masonry is also faster than that of the core-filled NC masonry. This is likely due to the toughening and crack-resistant effects of steel fibers, which make the mechanical properties of SFRC superior to those of NC at the same strength grade [36], thus further enhancing the overall compressive strength of the masonry. From Figure 5c, as the block wall thickness increases, the cross-sectional proportion of core-filled concrete decreases, which in turn leads to a gradual decline in the compressive strength of the masonry. This phenomenon is particularly prominent in the core-filled SFRC parameter group, indicating that core-filled SFRC has a more significant impact on the compressive strength of masonry. From Figure 5d, it can be seen that the compressive strength of masonry increases with the increase in steel fiber content in core-filled SFRC, but the growth amplitude is relatively limited. This indicates that an appropriate increase in steel fiber content is beneficial to improving the compressive strength of masonry. However, in practical engineering, attention should be paid to the optimal content of steel fibers to avoid construction issues such as fiber agglomeration caused by excessive fiber content [37].

The cross-sectional configuration diagram of the masonry is shown in Figure 1. When the bond-slip effect between the block and the core-filled concrete is not considered, according to the cross-sectional axial force equilibrium condition, the following can be known:

(14) $$f_{m}A=\left(A-A_{0}\right)f_{b}+A_{0}f_{g}$$

where *f_m_* is the compressive strength of MTGM, *f_b_* is the compressive strength of block concrete, *f_g_* is the compressive strength of grouted concrete, and *A* is the cross-sectional area, *A*_0_ is the grouting area.

In addition, the filling ratio of core-filled concrete is *γ* = *A*_0_/*A*. Therefore, the compressive strength of masonry can be further expressed as Equation (15):

(15) $$f_{m}=\left(1-\gamma \right)f_{b}+{\gamma f}_{g}$$

According to the Code for Design of Masonry Structures of China [38], the compressive strength of concrete blocks can be expressed as follows:

(16) $$f_{b}=0.368f_{bc}^{0.9}$$

where *f_bc_* is the axial compressive strength of block concrete.

Assuming that the compressive constitutive relations of core-filled NC and SFRC both follow the quadratic parabola model, the following is true:

(17) $$\sigma_{sc}=\left[2\left(\frac{\varepsilon }{\varepsilon_{sc}}\right)-2{\left(\frac{\varepsilon }{\varepsilon_{sc}}\right)}^{2}\right]f_{sc}$$

Since the bond-slip effect between the block and core-filled concrete is not considered, the peak strain of the masonry can be substituted into Equation (17), thus obtaining the strength exerted by the core-filled concrete when the masonry reaches the peak strain:

(18) $$f_{g}=\left[2\left(\frac{\varepsilon_{m}}{\varepsilon_{sc}}\right)-2{\left(\frac{\varepsilon_{m}}{\varepsilon_{sc}}\right)}^{2}\right]f_{sc}$$

The uniaxial compressive peak strain and compressive strength of SFRC are determined using Equations (7) and (8), respectively. These values are then substituted into Equation (18). Subsequently, the compressive strength of the masonry can be solved by incorporating Equations (14)–(18).

Based on the compressive strength prediction model proposed in this paper, fitting verification was conducted on the compressive strength of hollow block grouted masonry. Using the proposed model, GB 50003 code model [38], Zhu et al.’s model [39], Chen et al.’s model [40], Zhou et al.’s model [41], and Huang et al.’s model [42], the masonry compressive strength data provided in this study as well as in the studies by Yuan et al. [43], Zhou et al. [41], and Zhu et al. [39] were comprehensively compared. The comparison results are shown in Table 2 and Figure 5.

**Figure 6:**
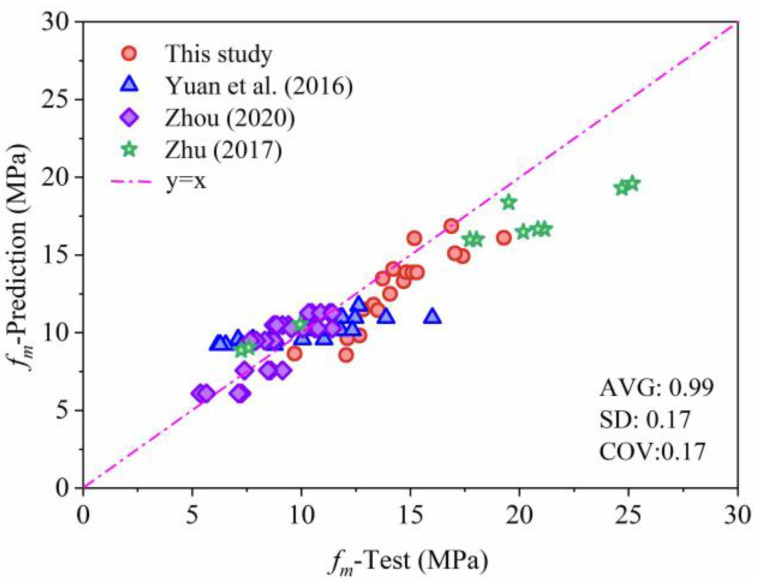
Fitting results for the axial compression model proposed in this paper [39,41,43].

Figure 6 shows the results of fitting the axial compression model. The results indicate that the coefficient of determination R^2^ of the proposed model is 0.99, with a coefficient of variation of 0.17. Compared with existing compressive strength prediction models for core-filled masonry, this model exhibits better performance in terms of both its prediction accuracy and discrete data control. This is mainly because the prediction model fully considers the strain development degree of core-filled concrete and incorporates the enhancement effect of steel fibers on the deformation capacity and strength of core-filled concrete.

### 3.4. Ultimate Strain

The ultimate strain (*ε*_cu_) of masonry in different parameter groups is defined as the strain corresponding to the stress dropping to 0.8*f*_m_ in the descending segment of the stress–strain curve. The ultimate strain of masonry exhibits an increasing trend with rising block strength. Overall, the ultimate strain of masonry with core-filled SFRC blocks is greater than that with core-filled NC blocks, but the difference diminishes as block strength increases. This indicates that increasing the strength grade of the blocks themselves enhances the ultimate deformation capacity of the masonry and mitigates the influence of different concrete grades in the core filling on the masonry’s ultimate deformation capacity. In practical engineering, the appropriate block strength grade should be selected based on a comprehensive evaluation of the masonry’s overall load-bearing performance and cost. The ultimate strain of the masonry increases with the strength grade of the core-filled concrete, particularly when SFRC is used, where the rate of increase is faster. This indicates that enhancing the strength of the core-filled concrete contributes to improving the masonry’s ultimate deformation capacity. Additionally, the presence of steel fibers further enhances the masonry’s ultimate deformation capacity. The ultimate strain of masonry decreases with increasing block wall thickness, with a more pronounced reduction observed for core-filled SFRC. Consistent with the influence patterns of compressive strength and peak strain, the core-filled area decreases as wall thickness increases, thereby reducing the contribution of core-filled SFRC to the ultimate deformation of masonry. As the steel fiber content increases, the ultimate strain of the masonry rapidly increases, further demonstrating that steel fibers have a more significant impact on the ultimate deformation of masonry. This is primarily due to the bridging effect between the steel fibers and the matrix [44], which delays the failure process of the masonry.

Based on the comprehensive analysis above, the factors affecting the ultimate strain of masonry include the strength grade of block concrete, the filling ratio of core-filled concrete, and the strength grade of core-filled concrete, and additionally the steel fiber content of core-filled concrete also has a significant impact on it. Therefore, combined with the laws presented in the data of this study, the ultimate strain of masonry can be expressed as a function of peak strain and steel fiber content of core-filled concrete, as Equation (19):

(19) $$\varepsilon_{u}=E\varepsilon_{m}^{\left(Fn_{f}+G\right)}$$

After nonlinear fitting analysis, the coefficients *E*, *F*, and *G* are obtained as 3.463, 3.256, and 0.824, respectively, with the coefficient of determination R^2^ = 0.961. Therefore, the ultimate strain of the masonry can be further expressed as Equation (20):

(20) $$\varepsilon_{u}=3.463\varepsilon_{m}^{\left(3.256n_{f}+0.824\right)}$$

### 3.5. Compressive Toughness

The compressive toughness of masonry can be characterized by the area under its compressive stress–strain curve before the peak. When NC is infilled in the masonry, increasing the strength of the block concrete has a negligible effect on the compressive toughness of the masonry. This is because ordinary concrete itself exhibits high brittleness with rapid crack propagation; after infilling, it mainly improves the compressive strength of the masonry but contributes little to its toughness. When SFRC is infilled in the masonry, the compressive toughness of the masonry is improved overall, and it exhibits a decreasing trend as the strength of the block concrete increases. This indicates that SFRC can significantly enhance the toughness of the masonry, but an excessively high strength of the block concrete will offset this advantage due to the increased material brittleness and the deterioration of interface performance. Compared with NC infilling, the compressive toughness of masonry with SFRC infilling is increased by 16% to 27% overall, and it increases with the increase in the strength grade of the infilled concrete. This suggests that increasing the strength of the infilled concrete is an effective approach to improve the compressive toughness of the masonry, with particularly significant effects when SFRC is used. When NC is infilled, the compressive toughness of the masonry remains almost unchanged as the wall thickness of the block increases. This may be due to the fact that the toughness is mainly controlled by the brittleness of the infilled NC. When SFRC is infilled, the compressive toughness of the masonry decreases as the wall thickness of the block increases. This is because thick-walled blocks change the cross-sectional proportion or filling ratio of the infilled SFRC, thereby reducing the toughening contribution of SFRC. As the steel fiber content increases from 0 to 1.6%, the compressive toughness of the masonry increases gradually. This indicates that steel fibers have a significant enhancing effect on the compressive toughness of the masonry. Therefore, infilling SFRC can significantly improve the compressive toughness of masonry, and its toughening effect is influenced by multiple factors; increasing the strength of the infilled concrete can enhance toughness, but excessively high block strength will weaken this effect due to increased brittleness, and increasing the wall thickness of the block will reduce the toughening contribution of SFRC. In contrast, the toughness of NC-infilled masonry is hardly affected by changes in block strength and wall thickness.

### 3.6. Development of a Compressive Constitutive Relationship

Research has been conducted on the mechanical properties and constitutive relationships of concrete block infilled masonry. As shown in Table 3, this paper selects four relatively classical compressive constitutive models for block infilled masonry, including the model by Dhanasekar et al. [45], the model by B. Powell et al. [46], the model proposed by Zhu [47], and the model proposed by Zhu [39].

Based on the characteristics of uniaxial compressive stress–strain curves and the mechanical failure process of mortise–tenon type grouted masonry, this paper selects a quadratic parabola for the ascending segment of the stress–strain curve and a straight line for the descending segment. This is similar to the model proposed by B. Powell et al. [46]. Specifically, it can be expressed as Equation (21):

(21) $$\sigma_{c}=\left\{\begin{array}{ll} \left[M\frac{\varepsilon_{c}}{\varepsilon_{m}}+N{\left(\frac{\varepsilon_{c}}{\varepsilon_{m}}\right)}^{2}\right]f_{m} & \left(0\leq \varepsilon_{c}\leq \varepsilon_{m}\right)\\ \left[1-\frac{\varepsilon_{c}-\varepsilon_{m}}{5\left(\varepsilon_{u}-\varepsilon_{m}\right)}\right]f_{m} & \left(\varepsilon_{m}\leq \varepsilon_{c}\leq \varepsilon_{u}\right) \end{array}\right.$$

**Figure 7:**
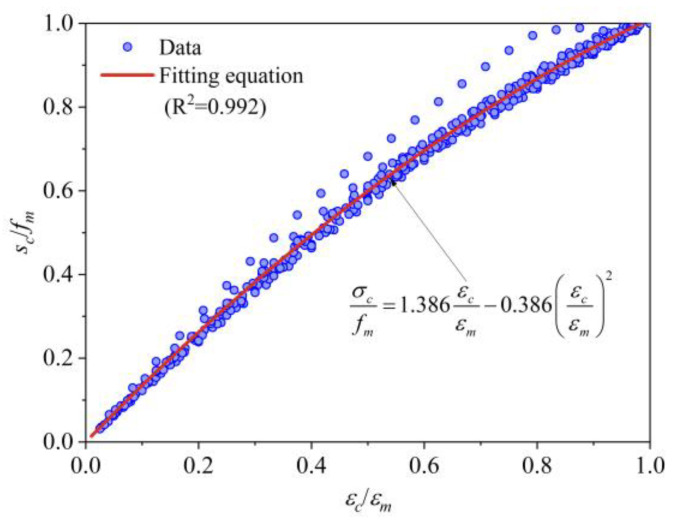
Fitting of the ascending segment of masonry compressive stress–strain curve.

Based on data fitting, the parameters M and N for the ascending segment are taken as 1.386 and −0.386, respectively, with R^2^ = 0.992, where the relevant details are shown in Figure 7. It should be noted that the ultimate strain of the masonry in this paper is taken as the strain corresponding to 0.8*f*_m_, which makes the descending segment of the stress–strain curve deterministic.

Based on curve fitting, a uniaxial compression constitutive relationship applicable to MTGM was proposed. The ascending section of this relationship is a quadratic parabola, and the descending section is linear:

(22) $$\sigma_{c}=\left\{\begin{array}{ll} \left[1.386\frac{\varepsilon_{c}}{\varepsilon_{m}}-0.386{\left(\frac{\varepsilon_{c}}{\varepsilon_{m}}\right)}^{2}\right]f_{m} & \left(0\leq \varepsilon_{c}\leq \varepsilon_{m}\right)\\ \left[1-\frac{\varepsilon_{c}-\varepsilon_{m}}{5\left(\varepsilon_{u}-\varepsilon_{m}\right)}\right]f_{m} & \left(\varepsilon_{m}\leq \varepsilon_{c}\leq \varepsilon_{u}\right) \end{array}\right.$$

**Figure 8:**
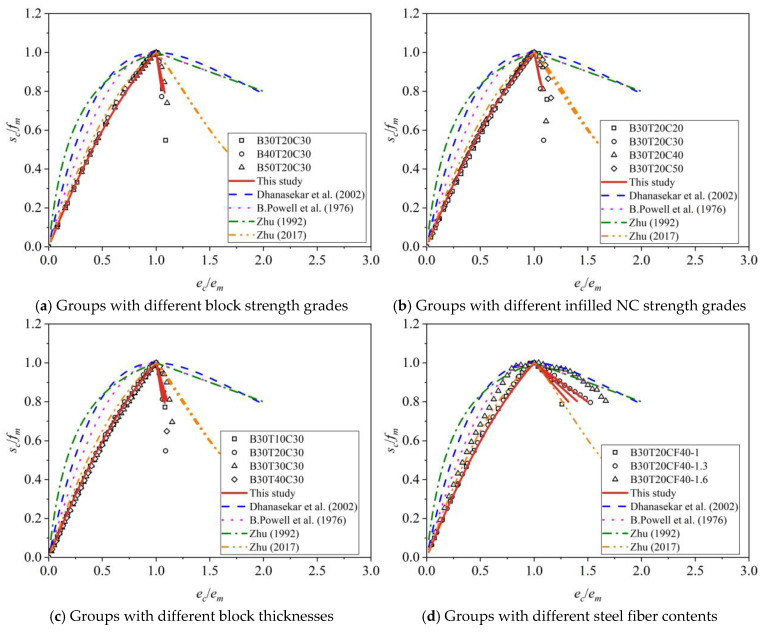
Comparison of compressive constitutive models in different parameter groups [39,45,46,47].

As shown in Figure 8, the proposed model is compared with four existing constitutive models. In the ascending branch, both the proposed model and B. Powell’s model [46] align well with the observed mechanical characteristics, confirming the suitability of a quadratic parabola for simulating the nonlinear compressive behavior. For the descending branch, however, the models by Dhanasekar [45], B. Powell [46], and Zhu [47] show significant deviations in predicting the slope. In contrast, the proposed model and Zhu’s model [39] provide a better fit. It should be noted that the mortise–tenon dry connection leads to a more brittle failure process compared to mortar-bonded masonry. Consequently, the ultimate strain in this analysis is defined as the point where compressive strength drops to 80%, a practical limitation not present in Zhu’s model [39]. While Zhu’s approach may be more flexible for nonlinear analysis, the present definition has greater practical relevance. Thus, the constitutive relationship developed in this study provides an optimized representation based on existing frameworks and accurately simulates the compressive behavior of infilled block masonry.

## 4. Simulation Results and Discussion on Eccentric Compression

### 4.1. Load–Displacement Curve

**Figure 9:**
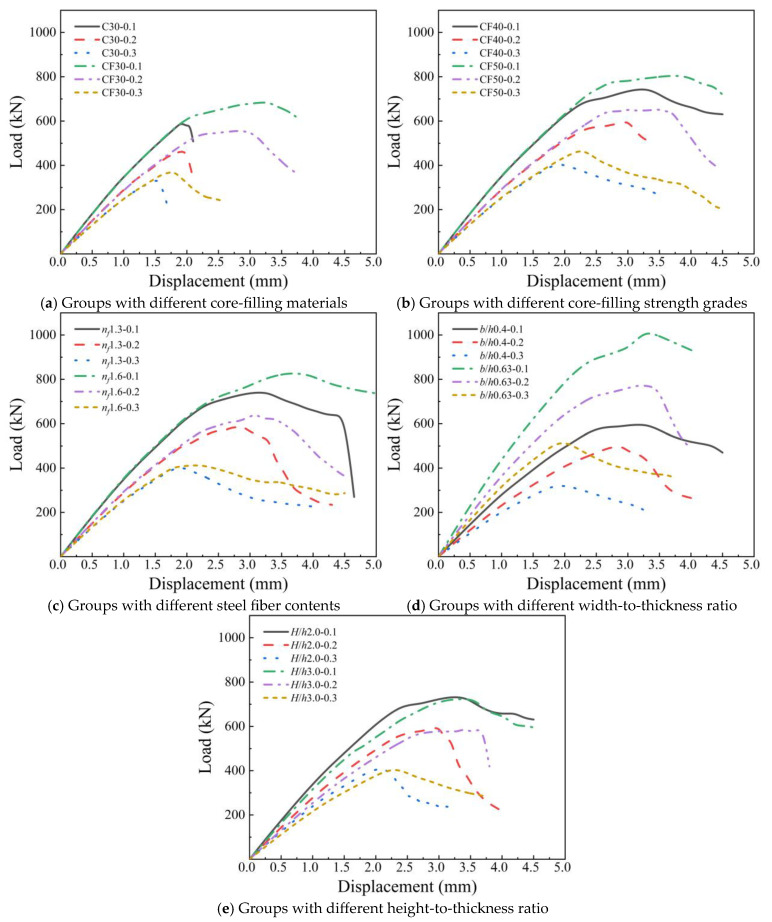
The load–displacement curves of models in different parameter groups.

To further investigate the mechanical behavior of tenon-and-mortise grouted masonry under eccentric compression and establish corresponding calculation methods, this section conducts a parametric numerical simulation study. As summarized in Table 4, a total of 33 numerical models are designed, with their dimensions and configuration based on the reference model detailed in Section 2.1. The concrete strength grade of the blocks is C30, with a wall thickness of 20 mm and an overall masonry thickness of 400 mm, while the remaining parameters are varied. The modeling methodology follows the procedures outlined in Section 2.1 Figure 9 shows the load–displacement curves of models under eccentric compression for each parameter group of masonry. Where C30 means the grouted concrete strength is 30 MPa, 0.1 means the eccentricity ratio (*e*/*h*) is 0.1, CF40 means the grouted steel fiber-reinforced concrete (SFRC) strength is 40 MPa, *n_f_* is the steel fiber volume fraction, *b*/*h* is the width-to-thickness ratio, and *H*/*h* is the height-to-thickness ratio.

Overall, as the eccentricity increases from 0.1 to 0.3, the peak load, peak displacement, and curve slope of the masonry in all parameter groups gradually decrease. This is because under eccentric compression, the cross-section is not only subjected to compressive force but also to an additional bending moment, resulting in uneven stress distribution across the cross-section. Tensile stress may occur on the side far from the external force, which in turn leads to the gradual degradation of the compressive performance of the masonry. It is worth noting that, when the core-filled NC is replaced with SFRC of the same strength grade, there is a significant difference in the descending branches of the curves: the curve of the NC-core-filled group drops more steeply, showing more obvious brittleness of the masonry; while the curve of the SFRC-core-filled group declines more gently, indicating the improved ductility of the masonry. This is mainly due to the fact that under the action of the additional bending moment, the SFRC in the masonry can fully exert its tensile performance. The bridging effect between steel fibers and the matrix inhibits the propagation of tensile cracks and mitigates the brittle failure process of the masonry. The laws of the other parameter groups are relatively similar to the compressive behavior of masonry under axial compression, and will not be elaborated here.

### 4.2. The Effect of Eccentricity on Compressive Bearing Capacity

**Figure 10:**
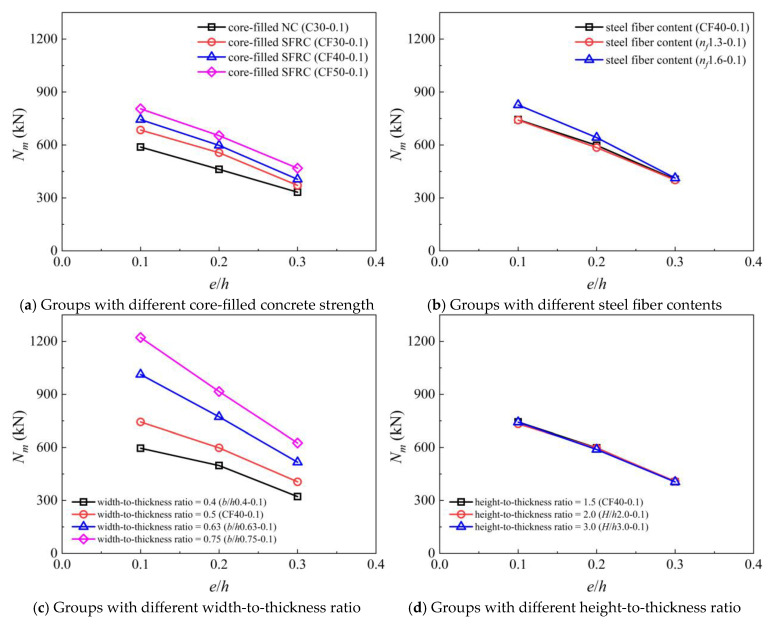
The effect of eccentricity on the compressive bearing capacity of models in different parameter groups.

Figure 10 illustrates the effect of eccentricity on the compressive bearing capacity (*N_m_*) of masonry under each parameter gradient. Overall, within the eccentricity range of 0.1–0.3, the compressive bearing capacity of masonry shows a linear decrease. As shown in Figure 10a, at the same eccentricity, core-filled SFRC and increasing the strength of core-filled concrete can improve the compressive bearing capacity of masonry, but with the increase in eccentricity, the degree of this improvement seems to decrease. This is mainly because under a relatively large eccentricity, part of the masonry’s cross-section may be in a tensile state, which accelerates the masonry’s failure process. Similar phenomena occur with other parameters, as shown in Figure 10b,c. Although the compressive bearing capacity of masonry increases with the increase in fiber content and width-to-thickness ratio, the magnitude of this improvement decreases with the increase in eccentricity. As shown in Figure 10d, at the same eccentricity, changing the height-to-thickness ratio (within the range of 1.5–3.0) has almost no effect on the compressive bearing capacity of the masonry. Within the studied height-to-thickness ratio of 3.0, the mortise–tenon grouted masonry can be classified as short columns, and instability effects are negligible. The behavior of long columns with a height-to-thickness ratio exceeding 3.0 requires further investigation.

### 4.3. Calculation Method for Eccentric Compression Bearing Capacity

#### 4.3.1. Basic Assumptions

Before conducting the eccentric compression calculation of mortise–tenon-type grouted masonry columns, the following four basic assumptions must be satisfied. (1) Under eccentric compression, the cross-section of the masonry complies with the plane section assumption. (2) The tensile stress–strain relationship of the masonry complies with the masonry compressive constitutive model (Equation (22)). Moreover, the condition for compressive failure of the masonry is that the compressive stress and compressive strain reach the peak stress and peak strain of the masonry. (3) Tensile strength is not considered for core-filled NC, while it is considered for core-filled SFRC. The tensile contribution of blocks and core-filled NC is ignored, whereas that of core-filled SFRC is taken into account. (4) The confinement effect of blocks on core-filled concrete is not considered.

#### 4.3.2. Derivation of Bearing Capacity Calculation Formula

**Figure 11:**
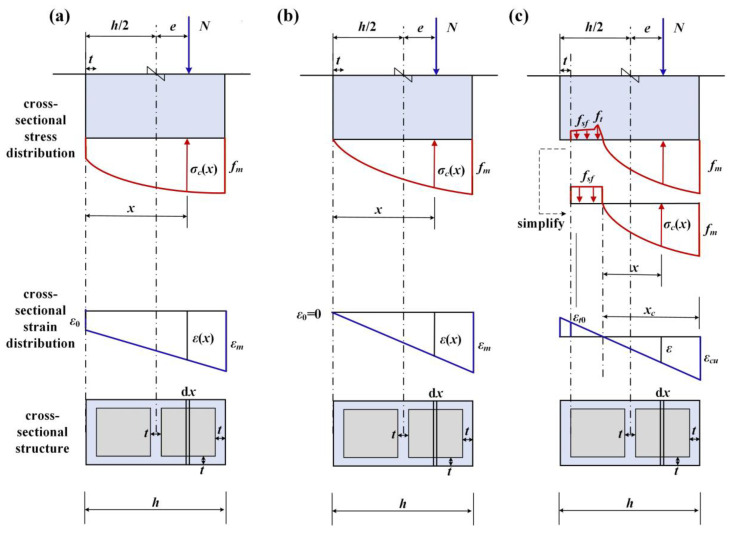
Calculation diagram of eccentric compression: (**a**) full cross-section compression; (**b**) critical state; and (**c**) cross-section under compression and tension.

The eccentric bearing capacity is derived based on the plane section assumption and the constitutive model established in Section 3.6. Three distinct stress scenarios, illustrated in Figure 11, are considered: full cross-section compression, the critical state, and combined tension–compression:

The first is the full cross-section compression condition. As shown in Figure 11a, when the eccentricity (*e*) is small, the entire cross-section of the masonry is in compression. The stress distribution of the cross-section is a curved trapezoid, and the strain distribution is a right trapezoid.

The second is the critical condition between full cross-section compression and partial cross-section tension. As shown in Figure 11b, when the eccentricity *e* increases to the critical eccentricity (*e_cr_*), the edge of the cross-section farthest from the external force is in a state of zero stress. At this time, the stress distribution of the cross-section is a curved triangle, and the strain distribution is a right triangle.

The third is the combined tension and compression condition of the cross-section. As shown in Figure 11c, when the *e* is greater than *e_cr_*, a certain range of the cross-section away from the external force is in a state of tension. At this time, the actual stress state of SFRC can be simplified to a rectangular stress distribution, where the tensile stress can be taken as the residual tensile strength (*f_sf_*) of SFRC. The stress distribution in the compressive zone of the cross-section remains a curved triangle, and the strain distribution of the entire cross-section is a diagonal triangle.

The governing equations for these scenarios are derived from cross-sectional equilibrium and are presented below.

(23) $$\varepsilon (x)=\varepsilon_{0}+\frac{\varepsilon_{m}-\varepsilon_{0}}{h}x$$

(24) $$N=\int_{0}^{h}\;\sigma (\varepsilon )b\;dx$$

(25) $$N=\int_{0}^{h}\;\left[1.386\frac{\varepsilon_{0}+\frac{\varepsilon_{m}-\varepsilon_{0}}{h}x}{\varepsilon_{m}}-0.386{\left(\frac{\varepsilon_{0}+\frac{\varepsilon_{m}-\varepsilon_{0}}{h}x}{\varepsilon_{m}}\right)}^{2}\right]bf_{m}\;dx$$

(26) $$\frac{N}{bhf_{m}}=0.564\frac{\varepsilon_{0}}{\varepsilon_{m}}+0.564-0.129{\left(\frac{\varepsilon_{0}}{\varepsilon_{m}}\right)}^{2}$$

(27) $$N\left(\frac{h}{2}+e\right)=\int_{0}^{h}\;\sigma (\varepsilon )xb\;dx$$

(28) $$\left.N\left(\frac{h}{2}+e\right)=\int_{0}^{h}\;\left[1.386\frac{\varepsilon_{0}x+\frac{\varepsilon_{m}-\varepsilon_{0}}{h}x^{2}}{\varepsilon_{m}}-0.386{\left(\frac{\varepsilon_{0}+\frac{\varepsilon_{m}-\varepsilon_{0}}{h}x}{\varepsilon_{m}}\right)}^{2}\right]x\right]bf_{m}\;dx$$

(29) $$\frac{N\left(\frac{h}{2}+e\right)}{bh^{2}f_{m}}=0.167\frac{\varepsilon_{0}}{\varepsilon_{m}}+0.366-0.032{\left(\frac{\varepsilon_{0}}{\varepsilon_{m}}\right)}^{2}$$

(30) $$e_{cr}=0.15h$$

(31) $$\varepsilon (x)=\frac{\varepsilon_{m}}{x_{c}}x$$

(32) $$N=\int_{0}^{x_{c}}\;\sigma (\varepsilon )b\;dx-\left(h-x_{c}-t\right)(b-2t)f_{sf}$$

(33) $$N=0.564bx_{c}f_{m}-\left(h-x_{c}-t\right)(b-2t)f_{sf}$$

(34) $$N\left(\frac{h}{2}+e\right)=\int_{0}^{x_{c}}\;\sigma (\varepsilon )\left(h-x_{c}+x\right)b\;dx-\left(h-x_{c}-t\right)(b-2t)f_{sf}\frac{h-x_{c}+t}{2}$$

(35) $$N\left(\frac{h}{2}+e\right)=\left(0.564x_{c}h-0.198x_{c}^{2}\right)bf_{m}-\left(h-x_{c}-t\right)\left(h-x_{c}+t\right)(b-2t)\frac{f_{sf}}{2}$$

For the full compression scenario (Figure 11a), the axial load *N* is determined by solving the system of Equations (26) and (29). The critical eccentricity for the transition state (Figure 11b) is given directly by Equation (30). For the combined tension–compression scenario (Figure 11c), the compressive zone depth *x_c_* and the axial load *N* are obtained by solving Equations (33) and (35). The calculated eccentric bearing capacities for all numerical models using this methodology are summarized in Table 4.

#### 4.3.3. Comparison and Verification of Calculation Results

**Figure 12:**
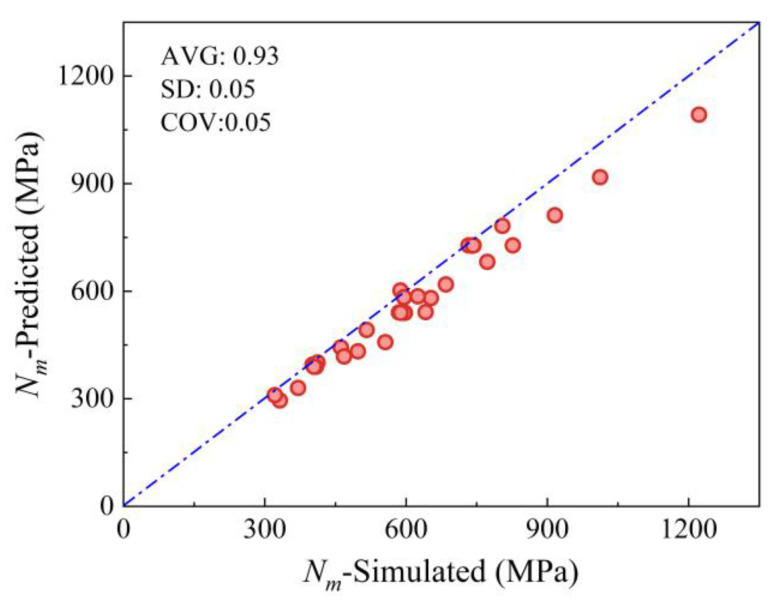
Fitting results for the eccentric compression model proposed.

As shown in Figure 12, the proposed model achieves a prediction accuracy of 0.93 and a dispersion coefficient of 0.05. This improvement stems from the establishment of a compressive constitutive model for the MTGM, which fully accounts for its structural and load-bearing characteristics.

To facilitate design applications, a simplified formula for the bearing capacity under eccentric compression was derived through nonlinear regression of theoretical results:

(36) $$N=\left(1-3.76\frac{e}{h}+5.38{\left(\frac{e}{h}\right)}^{2}\right)bhf_{m}$$

**Figure 13:**
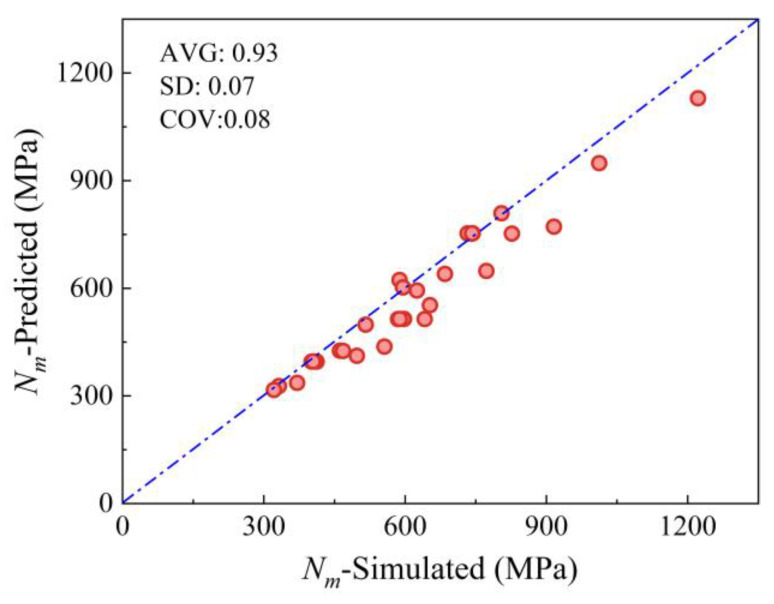
Fitting results for the simplified eccentric compression model proposer.

The results obtained using Equation (36) are shown in Figure 13, exhibiting an accuracy of 0.93 and a dispersion coefficient of 0.08. The simplified computational model established based on the method proposed herein remains capable of reliably predicting the eccentric compression capacity of MTGM.

## 5. Practical Implications and Limitations

### 5.1. Implications for Cost and Constructability

The performance advantages of MTGM directly translate into several clear economic and construction benefits. First, the mortarless, dry-stack assembly eliminates all material and labor costs for mortar bedding and curing, which can account for approximately 15–25% of traditional masonry wall cost. This leads to faster construction cycles, with dry-stack methods typically reporting 20–40% reduction in assembly time. Second, our results show that even 1% steel fiber content can increase ultimate strain by over 20%, and 1.6% content can increase it by approximately 37%, significantly enhancing damage tolerance and potentially reducing long-term maintenance. Thus, the MTGM system presents a compelling value proposition where the initial investment in high-performance materials is balanced by savings in construction speed, labor, and improved lifecycle resilience.

### 5.2. Limitations and Future Research Directions

While this study provides comprehensive insights into the compressive behavior of MTGM, several limitations should be acknowledged to contextualize the findings. First, the numerical model assumes perfect bonding between the grout core and the block, neglecting potential bond-slip behavior that might occur under extreme deformation or with different interfacial textures. Second, the material constitutive models, though validated, employ simplified homogeneous properties and do not account for the stochastic nature of concrete cracking or the anisotropic distribution of steel fibers. Third, the parametric study, while extensive, is confined to a specific geometric configuration of the mortise-tenon joint; the influence of key geometric parameters on performance remains to be quantified. Fourth, all analyses are based on monotonic loading; the cyclic or seismic performance, which is critical for structural application, requires separate investigation. Finally, all analyses in this study are based on specimens with a height-to-thickness ratio not exceeding 3.0, which are considered short columns where material strength governs the failure mode. The implications of this choice are twofold: (1) the developed constitutive and bearing capacity models are validated within this regime and may not be directly applicable to slender columns, where buckling instability and second-order effects become predominant; (2) the plane section assumption, which underpins the eccentric compression analysis, holds well for short columns but may require modification for slender members. Consequently, the application of the proposed design methods in practice should be limited to walls and columns with comparable slenderness, or complemented by stability checks as per relevant design codes.

These limitations, however, do not undermine the core findings regarding the parametric trends and the proposed models within the defined scope. Rather, they outline clear pathways for future work: (1) experimental and numerical studies on the grout–block interface behavior, including bond-slip; (2) high-fidelity modeling incorporating material heterogeneity and damage stochasticity; (3) parametric optimization of the mortise-tenon joint geometry; (4) cyclic and dynamic load testing coupled with numerical simulation; and (5) stability analysis and model extension for slender MTGM members.

## 6. Conclusions

This study systematically investigated the compressive performance of mortise-and-tenon grouted masonry (MTGM) through integrated numerical simulation and theoretical analysis. The key findings are summarized as follows:

1. A refined three-dimensional finite element model was established and rigorously validated against experimental results. This model accurately reproduced complex failure modes and load–displacement responses, with an average peak load ratio of 0.96 between simulated and experimental values, establishing a reliable foundation for parameter analysis.
2. Compressive strength increases with higher block and grout strengths; the C50 grout group exhibited approximately 56.7% higher strength than the C20 group. Using steel fiber-reinforced concrete as grout significantly enhances ductility and toughness, with increasing fiber content from 1% to 1.6% raising ultimate strain by about 37.0%. Reducing the block wall thickness from 40 mm to 10 mm increases the grout’s contribution, boosting masonry compressive strength by 13.8% to 39.4%.
3. The developed axial compressive strength calculation formula outperforms existing models, achieving a high coefficient of determination (R^2^ = 0.99) and low dispersion. A corresponding constitutive model with a parabolic ascending branch and a linear descending branch was also established, exhibiting excellent data fit (R^2^ = 0.992).
4. The bearing capacity decreases approximately linearly with increasing eccentricity. When the eccentricity ratio increases from 0.1 to 0.3, the average reduction is approximately 40%. The masonry infill of steel fiber-reinforced concrete maintains good ductility even at high eccentricity ratios. The simplified design formula derived based on the plane section assumption provides reliable predictions with an accuracy of 0.93.

In summary, this study provides validated numerical insights, precise predictive models, and practical design methods for the MTGM system. These results establish a solid foundation for its structural applications and design optimization.

## Figures and Tables

**Table 1:** The value of the coefficient for steel fiber concrete.

Steel Fiber Concrete	*α_c_*	*β*	*α_t_*
CF30	0.459	0.759	0.76
CF40	0.25	0.478	
CF50	0.142	0.416	

**Table 2:** Comparison of compressive strength prediction models for different hole-grouted block masonry.

Models	Type	AVG	COV	References
$f_{m,GB}=(1-\gamma )f_{b}+0.94\gamma f_{sc}$	-	1.19	0.29	GB [38]
$f_{m,Zhu}=\frac{1.1f_{sc}^{2}+1.78f_{sc}f_{b}+0.58f_{b}^{2}}{3.83f_{sc}+0.92f_{b}}$	Concrete block core-filled NC masonry	0.93	0.32	Zhu [39]
$f_{m,Chen}=1.54(1-\gamma )f_{b}+0.947\gamma f_{sc}$	EPS–cotton straw fiber hole-grouted lightweight masonry	1.00	0.40	Chen [40]
$f_{m,\mathrm{Zhou}}=\left(0.66+0.068\frac{f_{\mathrm{sc}}}{f_{bc}}\right)\left(0.62f_{bc}+0.38f_{mortar}\right)$	Mortise–tenon type hole-grouted NC masonry	0.71	0.31	Zhou [41]
$f_{m,\mathrm{Huang}}=0.368f_{b0}^{0.9}(1-\gamma )+0.81f_{sc}$	Mortar-free hole-grouted NC masonry	1.09	0.46	Huang [42]

**Table 3:** Compressive constitutive model of core-filled block masonry.

Models	References
$\frac{\sigma }{f_{m}}=\frac{4\varepsilon /\varepsilon_{m}}{1.5\left(1+\varepsilon /\varepsilon_{m}\right)+{\left(\varepsilon /\varepsilon_{m}\right)}^{2.5}}$	Dhanasekar [45]
$\frac{\sigma }{f_{m}}=\left\{\begin{array}{l} 2\varepsilon /\varepsilon_{m}-{\left(\varepsilon /\varepsilon_{m}\right)}^{2}\;\left(0\leq \varepsilon \leq \varepsilon_{m}\right)\\ 1.2-0.2\varepsilon /\varepsilon_{m}\;\;\left(\varepsilon_{m}\leq \varepsilon \leq 1.6\varepsilon_{m}\right) \end{array}\right.$	B. Powell [46]
$\frac{\sigma }{f_{m}}=\left\{\begin{array}{l} \frac{\varepsilon /\varepsilon_{m}}{0.2+0.8\varepsilon /\varepsilon_{m}}\;\left(0\leq \varepsilon \leq \varepsilon_{m}\right)\\ 1.2-0.2\varepsilon /\varepsilon_{m}\;\;\left(\varepsilon_{m}\leq \varepsilon \right) \end{array}\right.$	Zhu [47]
$\frac{\sigma }{f_{m}}=\left\{\begin{array}{l} 1.6\varepsilon /\varepsilon_{m}-0.6{\left(\varepsilon /\varepsilon_{m}\right)}^{2}\;\;\left(0\leq \varepsilon \leq \varepsilon_{m}\right)\\ k_{1}\frac{0.82-0.18\varepsilon /\varepsilon_{m}}{{\left(\varepsilon /\varepsilon_{m}\right)}^{2}-k_{0}\varepsilon /\varepsilon_{m}+1.64}\;\left(\varepsilon_{m}\leq \varepsilon \right) \end{array}\right.$	Zhu [39]

Note: *k*_0_ is the adjustment coefficient for the descending segment of the curve, with the expression *k*_0_ = 0.44*f*_m_^0.5^; *k*_1_ is the adjustment coefficient for the peak value, with the expression *k*_1_ = 4.13 − 1.56 × *k*_0_.

**Table 4:** Simulated variables and analysis results for eccentric compression of MTGM.

Group	*f_f_*/MPa	*n_f_*/%	*b*/*h*	*H*/*h*	*e*/*h*	*N_m_*/kN	*δ_m_*/mm	*N_mp_*/kN	*N_mp_*/*N_m_*
C30-0.1	C30	0	0.5	1.5	0.1	588	1.90	602	1.02
C30-0.2	C30	0	0.5	1.5	0.2	462	1.90	443	0.96
C30-0.3	C30	0	0.5	1.5	0.3	332	1.50	295	0.89
CF30-0.1	CF30	1	0.5	1.5	0.1	685	3.30	619	0.90
CF30-0.2	CF30	1	0.5	1.5	0.2	556	2.85	458	0.82
CF30-0.3	CF30	1	0.5	1.5	0.3	371	1.80	330	0.89
CF40-0.1	CF40	1	0.5	1.5	0.1	744	3.30	728	0.98
CF40-0.2	CF40	1	0.5	1.5	0.2	598	3.00	540	0.90
CF40-0.3	CF40	1	0.5	1.5	0.3	405	1.95	389	0.96
CF50-0.1	CF50	1	0.5	1.5	0.1	805	3.75	782	0.97
CF50-0.2	CF50	1	0.5	1.5	0.2	653	3.50	581	0.89
CF50-0.3	CF50	1	0.5	1.5	0.3	469	2.25	418	0.89
*n_f_*1.3-0.1	CF40	1.3	0.5	1.5	0.1	741	3.15	727	0.98
*n_f_*1.3-0.2	CF40	1.3	0.5	1.5	0.2	585	2.90	541	0.92
*n_f_*1.3-0.3	CF40	1.3	0.5	1.5	0.3	402	1.90	395	0.98
*n_f_*1.6-0.1	CF40	1.6	0.5	1.5	0.1	827	3.75	728	0.88
*n_f_*1.6-0.2	CF40	1.6	0.5	1.5	0.2	642	3.10	542	0.84
*n_f_*1.6-0.3	CF40	1.6	0.5	1.5	0.3	412	2.10	401	0.97
*b*/*h*0.4-0.1	CF40	1	0.4	1.5	0.1	596	3.15	583	0.98
*b*/*h*0.4-0.2	CF40	1	0.4	1.5	0.2	498	2.90	432	0.87
*b*/*h*0.4-0.3	CF40	1	0.4	1.5	0.3	322	1.95	310	0.96
*b*/*h*0.63-0.1	CF40	1	0.63	1.5	0.1	1012	3.30	918	0.91
*b*/*h*0.63-0.2	CF40	1	0.63	1.5	0.2	773	3.20	682	0.88
*b*/*h*0.63-0.3	CF40	1	0.63	1.5	0.3	517	1.95	492	0.95
*b*/*h*0.75-0.1	CF40	1	0.75	1.5	0.1	1222	3.60	1092	0.89
*b*/*h*0.75-0.2	CF40	1	0.75	1.5	0.2	916	3.30	812	0.89
*b*/*h*0.75-0.3	CF40	1	0.75	1.5	0.3	625	1.95	586	0.94
*H*/*h*2.0-0.1	CF40	1	0.5	2.0	0.1	733	3.30	728	0.99
*H*/*h*2.0-0.2	CF40	1	0.5	2.0	0.2	596	3.00	540	0.91
*H*/*h*2.0-0.3	CF40	1	0.5	2.0	0.3	409	2.10	389	0.95
*H*/*h*3.0-0.1	CF40	1	0.5	3.0	0.1	742	3.45	728	0.98
*H*/*h*3.0-0.2	CF40	1	0.5	3.0	0.2	589	3.40	540	0.92
*H*/*h*3.0-0.3	CF40	1	0.5	3.0	0.3	405	2.25	389	0.96

## Data Availability

The original contributions presented in this study are included in the article. Further inquiries can be directed to the corresponding authors.

## Nomenclature

G*_f_*	The tensile fracture energy
G*_c_*	The compressive fracture energy
*f*_c_	Axial compressive strength of concrete
*ε*_0_	The elastic strain
*ε*_c_	The peak strain
*ε*_u_	The ultimate strain
*E*	Elastic modulus of concrete
*G*_sc_	The compressive fracture energy of SFRC
*f*_sc_	The compressive strength of SFRC
*n_f_*	The volume content of steel fibers
*l_f_*	The length of steel fibers
*d_f_*	The diameter of steel fibers
*ε*_sc_	The compressive peak strain of SFRC
*f*_st_	The uniaxial tensile strength of SFRC
*f*_t_	The tensile strength of concrete
*F_be_*	The shape factor of steel fibers
*f*_stu_	Th ultimate tensile strength of SFRC
*f_R,4_*	The ultimate flexural tensile strength
*E_p-c_*	The average elastic modulus of the steel plate and concrete
*l_e_*	The average size of elements near the interface
*ε*_m_	The peak strain of MTGM
*ε*_b_	The peak strain of block concrete
*γ*	The filling ratio of core-filled concrete
*A*	The cross-sectional area
*A*_0_	The grouting area
*f*_m_	The compressive strength of MTGM
*f*_b_	The compressive strength of block concrete
*f*_g_	The compressive strength of grouted concrete
*ε*_cu_	The ultimate strain
*f*_bc_	The axial compressive strength of block concrete
